# MicroRNAome of Porcine Pre- and Postnatal Development

**DOI:** 10.1371/journal.pone.0011541

**Published:** 2010-07-12

**Authors:** Mingzhou Li, Youlin Xia, Yiren Gu, Kai Zhang, Qiulei Lang, Lei Chen, Jiuqiang Guan, Zonggang Luo, Haosi Chen, Yang Li, Qinghai Li, Xiang Li, An-an Jiang, Surong Shuai, Jinyong Wang, Qi Zhu, Xiaochuan Zhou, Xiaolian Gao, Xuewei Li

**Affiliations:** 1 Institute of Animal Genetics and Breeding, College of Animal Science and Technology, Sichuan Agricultural University, Ya'an, Sichuan, China; 2 Department of Biology and Biochemistry, University of Houston, Houston, Texas, United States of America; 3 Department of Biology, Zhejiang University, Hangzhou, Zhejiang, China; 4 LC Sciences, Houston, Texas, United States of America; 5 Chongqing Academy of Animal Science, Chongqing, China; 6 School of Life Science, University of Science and Technology of China, Hefei, Anhui, China; Institute of Preventive Medicine, Denmark

## Abstract

The domestic pig is of enormous agricultural significance and valuable models for many human diseases. Information concerning the pig microRNAome (miRNAome) has been long overdue and elucidation of this information will permit an atlas of microRNA (miRNA) regulation functions and networks to be constructed. Here we performed a comprehensive search for porcine miRNAs on ten small RNA sequencing libraries prepared from a mixture of tissues obtained during the entire pig lifetime, from the fetal period through adulthood. The sequencing results were analyzed using mammalian miRNAs, the precursor hairpins (pre-miRNAs) and the first release of the high-coverage porcine genome assembly (Sscrofa9, April 2009) and the available expressed sequence tag (EST) sequences. Our results extend the repertoire of pig miRNAome to 867 pre-miRNAs (623 with genomic coordinates) encoding for 1,004 miRNAs, of which 777 are unique. We preformed real-time quantitative PCR (q-PCR) experiments for selected 30 miRNAs in 47 tissue-specific samples and found agreement between the sequencing and q-PCR data. This broad survey provides detailed information about multiple variants of mature sequences, precursors, chromosomal organization, development-specific expression, and conservation patterns. Our data mining produced a broad view of the pig miRNAome, consisting of miRNAs and isomiRs and a wealth of information of pig miRNA characteristics. These results are prelude to the advancement in pig biology as well the use of pigs as model organism for human biological and biomedical studies.

## Introduction

MicroRNAs (miRNAs), a well-defined group of small RNAs of ∼22 nucleotides (nt), are derived from ∼70 nt long stem-loop precursors (pre-miRNAs) located in all mammalian autosomes and X chromosome [1], [2]. miRNAs are now considered a major group of functional non-coding RNAs (ncRNAs) which function by pairing, albeit imperfectly, to mRNAs resulting, in many cases, in target-specific post-transcriptional repression [3], [4]. A single miRNA may potentially target hundreds of mRNAs [5]–[7]. The broad functional roles of miRNAs are still being elucidated.

*Sus scrofa* (i.e., pig or swine) was among the first animals to be domesticated ∼9,000 years ago [8] and is closely comparable to human in size, anatomy, physiology, metabolism, pathology and pharmacology [9]. The pig's usefulness has been expanding beyond being a protein source to becoming an important model system for human health, such as a future source of organs, tissues and cells for xenotransplantation [10]. Although predictably important, the microRNAome (miRNAome) of pig has been largely undefined. The latest miRBase 14.0 (September 2009) reports total 10,883 pre-miRNAs, but only 77 of these pre-miRNAs are from pig, which code for 72 distinct miRNAs and one miRNA* (miR-140*-3p), which originates from the hairpin pre-miRNA arm opposite to the annotated miRNA containing arm [11]. These numbers are unchanged from the previous version of miRBase (v13.0, March 2009). Thus far, most porcine miRNAs have been identified via *in silico* approaches with a small portion of these validated in limited experiments [12]–[15]. The number of porcine pre-miRNAs (77) thus far identified is much lower than those identified in species of similar genome sizes, such as human (721), chimpanzee (606), macaque (485), rat (325), mouse (579), cow (615), or even dog (321).

The pig genome is estimated at about 2.7 billion base pairs (Bbp) in size and is closer to human than rodent species. The economic and biomedical importance of the pig has led to significant efforts to decode the pig genome and its genetic components [8], [16]. The pig whole genome sequencing project was launched in early 2006 [17]. More recently, the high-coverage draft of pig genome (∼2.26 Bbp, ∼98% complete) was reported in November 2009. The availability of the nearly complete pig genome provides the opportunity to map its miRNAome and further define the function of these key molecules such as mRNAs and miRNAs in cellular regulatory networks.

We present herein a global survey of the pig miRNAome using deep sequencing technology. The ten small RNA libraries used in this study were culled from tissue samples representing ten developmental stages: six prenatal stages (30, 45, 60, 75, 90 and 105 days after insemination, hereafter referred to as E30d, E45d, E60d, E75d, E90d and E105d, respectively) and four postnatal stages (0, 30, 120 and 180 days after birth, hereafter referred to as Birth, 30 d, 120 d and 180 d, respectively). These time points cover major morphological and physiological changes of pig growth and development throughout pregnancy (∼114 days) and up to 180 days after birth when the pigs reach peak commercial value. Each small RNA library was sequenced individually using an Illumina sequencing instrument and the sequencing generated ∼9.4 million (M) 36-nt sequence reads resulting in a total of greater than 93.6 million sequence reads. Analysis of the sequencing data was performed primarily by using ACGT101-miR, an in-house program for discovering miRNAs from deep sequencing data (to be published separately).

## Results

### Sequencing data

Detailed information about the samples is presented in Tables S1 and S2. The overall flow of the sequencing data analysis is shown schematically in Figure S1. In the following discussions, the copy numbers of the sequenced sequences (sequ-seqs) are referred as “counts”; the sequ-seqs are also categorized as “kinds of sequences” and each kind of sequence contains identical sequ-seqs of one to multiple counts. The grouping of the sequ-seqs results in two types of clusters (Text S1). One type is a position-based cluster in which sequ-seqs are located within 50 nt by genomic positions. The second type is a sequence-based cluster in which sequ-seqs are related by certain sequence patterns.

Table S3 provides statistics (by counts and by kinds) of the high-copy (counts ≥3) as well as low-copy (counts <3) sequ-seqs. Each library has an average of 3.1 M counts and 0.4 M kinds of sequ-seqs before the filtering the data by copy numbers. We found that on average the sequ-seqs of higher counts (≥3) account for 78.2% of all sequ-seqs counts (2.7 M per library) but only about 7.2% by kinds of sequ-seqs (32.7 K per library). This means that fewer kinds of sequ-seqs dominate the population distribution of miRNA candidates. Given the low percentage of counts for the low-copy groups and the need for replicates due to their higher possibility of sequencing errors, these low-copy group sequ-seqs were excluded in the following data analysis. Table S3 also shows that the counts/kinds ratios for the low- and high-copy groups are 1.1 and 82.2, respectively. The high counts/kinds ratio for the high-copy sequ-seqs (counts ≥3) used for the following analyses ensured higher reliability of the reported results.

In our reported data, certain known types of RNA sequences (e.g., mRNA, rRNA, tRNA, snRNA, snoRNA and repetitive sequence elements), were excluded from analysis and these results are summarized in Table S4. These sequences account for only a small fraction (9.8%) of the kinds of sequ-seqs accounted for 1.1% in counts of all sequ-seqs. The detection of a low proportion of long RNAs, such as mRNA (1.1% by kinds and 0.2% by counts) and rRNA (1.5% by kinds and 0.2% by counts) indicates that the sequencing samples were not contaminated by degraded RNA, and are therefore of high integrity.

In the following description, we use the most abundant sequence and its count to represent a family of sequences that vary by length and/or vary by one nucleotide. For temporal trends and comparisons, we controlled sample amounts from tissues of the same mixture at each time point.

### Mappable sequences

**Figure 1:**
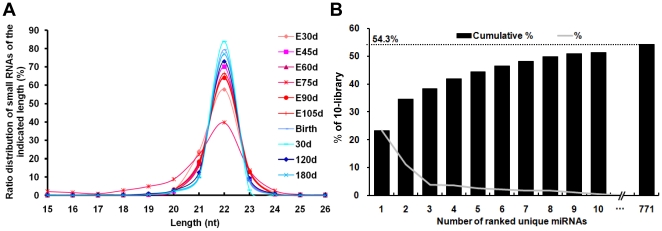
The length and counts characteristics of the sequencing results. (A) The length distribution of sequenced small RNAs. The red-pinkish colors indicate prenatal samples and blue colors indicate postnatal samples. (B) Plot of the unique miRNAs (starting from the miRNA with the highest counts, x-axis) versus their accumulative % in total counts of the mappable sequences. The dashed horizontal line at 54.3% represents the level of the total 771 unique miRNAs and the % of individual miRNA is marked by the gray line.

The raw sequ-seqs were passed through the digital filters of base-call quality using Illumina's Genome Analyzer Pipeline software, and sequence patterns (simple sequence compositions), length, copy number and other known RNA classes were analyzed using ACGT101-miR program and the resulting sequence are referred to as “mappable sequences”. Of these mappable sequences, the majority of the small RNAs are 20–24 nt in size, which is typical of small RNA of Dicer-processing products (Figure 1A). The predominant species in all ten libraries we surveyed were 22 nt small RNAs. We noticed a change in the length distribution width of the sequences, which narrows during the transition from the pre- to postnatal stages. The prenatal miRNAs show an increased number of sequences in 21 and 23 nt lengths, whereas the postnatal miRNAs converge to 22 nt lengths. The length variations of miRNA homologs have been repeatedly reported in deep sequencing of miRNAs of several species. These length variations have been mainly attributed to enzymatic modification such as RNA editing [18], 3′-editing [19], exonuclease activities [20], [21], etc. Presently, there is little understanding of the functional role of these homologs of highly abundant miRNAs. Our results show that the pattern of the miRNA length variation may not be constant during pig development. Further data collection and analysis are required to interpret this phenomenon.

The statistics of the reduced pool of mappable sequences are also summarized in Table S5 in terms of counts (copy numbers of all sequences) and kinds of sequences (identical sequences).

### Mapping and cataloging porcine miRNAs

We frequently observed sequence-heterogeneity at the 3′ end of the sequences and less heterogeneity at the 5′-end of the sequences. Based on the sequencing data it appears that multiple mature variants are produced. These sequences are named isomiRs as reported in literatures [22]–[25]. These isomiRs of miRNA were expressed at a range of levels. The existence of isomiRs has been commonly reported in miRNA studies using different platform technologies, suggesting that isomiRs are less likely due to artifacts created from deep sequencing technologies [26]. In these cases, the most frequently observed isomiR was chosen as a reference sequence. This provides the most robust approach for evaluation of differential expression. The following discussions in general refer to the most abundant isomiR (as reflected by the total counts over ten libraries), which perfectly matches the genome and expressed sequence tag (EST) or pre-miRNA sequences have been preferentially chosen.

As shown in the data processing workflow (Figure S1), the mature miRNA transcripts identified from the mappable sequences are divided into five groups: (1) 89 miRNAs corresponding to 60 known porcine pre-miRNAs which are also mapped to genome and EST. Specifically, 60 miRNAs are known but 29 were not previously identified (and thus new) porcine miRNA*s (Tables S6 and S7); (2) 19 miRNAs, which correspond to 12 known porcine pre-miRNAs, which cannot be mapped to genome or EST. Specifically, 11 miRNAs and one miRNA* (miR-140*-3p) are known in miRBase, and seven were not previously identified (and thus new) porcine miRNA*s (Tables S6 and S7); (3) 186 miRNAs corresponding to 139 other known miRBase mammalian pre-miRNAs which are mapped to the genome or EST. These are labeled PN(a) (porcine novel, “a” type) (Tables S8 and S9) and were previously unknown as porcine miRNAs/pre-miRNAs; (4) 211 miRNAs corresponding to 169 other known miRBase mammalian pre-miRNAs labeled PN(b) (porcine novel, “b” type) which cannot be mapped to the genome or EST (Tables S10 and S11); and (5) 493 miRNAs corresponding to 482 candidate pre-miRNAs, which are predicted RNA hairpins derived from genome and EST, and are labeled PC (porcine candidate) (Tables S12 and S13).

Table S14 provides a complete sequence, name, and relative abundance list for the 771 unique miRNAs (i.e., the most abundant isomiR) detected in this study and six known porcine miRNAs that were not detected in our ten libraries. Predictably, there exist identical mature sequences that originate from distinct pre-miRNAs and genomic loci, which result in these 771 unique miRNA sequences corresponding to 998 miRNAs and miRNA*s, which originating from 862 pre-miRNAs. In accordance with the miRNA naming conventions adopted by miRBase, these identical miRNAs from different pre-miRNAs were distinguished by the suffix such as ssc-miR-9-1 and ssc-miR-9-2 [11]. In the groups of cataloged miRNAs, we similarly observed that the majority of abundant miRNAs are from fewer miRNAs. As shown in Figure 1B, the top ten unique miRNAs with the highest expression level account for 51.4% (by counts) versus 54.3% (dashed line) by the total counts of all 771 unique miRNAs of the mappable sequences in all ten libraries. These miRNAs, expressed at high levels throughout all stages of the development from fertilization to adult, may be involved in basic functions needed for the sustenance of the cell cycle.

### The known porcine miRNAs

The majority of the mappable sequences (1.8 M counts, 75.0%) can be mapped to the 77 known porcine pre-miRNAs for each library. Furthermore, 73.3% and 1.7% of the sequ-seq counts are mapped or not mapped to genome and EST, respectively, and these represent 2,075 (19.1%) and 207 (2.0%) in kinds of different sequences for each library, respectively (Table S5). These results indicate that the high-abundant, known porcine miRNAs were more easily detected, but a far larger number of kinds of miRNAs from a much more diverse pool were not found until this study.

**Figure 2:**
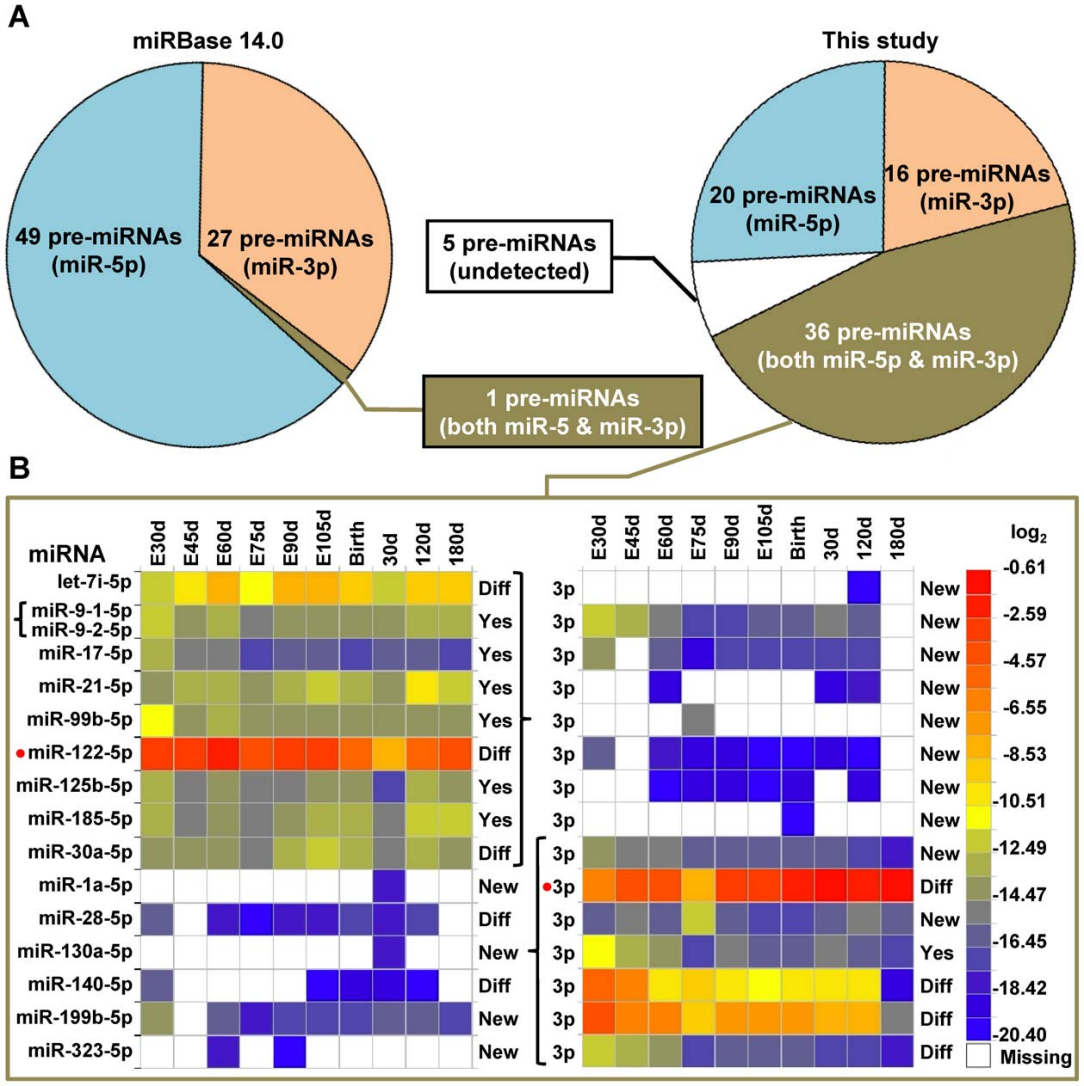
The temporal profile of the known porcine miRNAs and new miRNA*s. (A) The total pre-miRNAs represented in miRBase (left) and an updated count by this study (right). The pre-miRNAs in four groups: type containing miR-5p (blue), miR-3p (tan), both miR-5p and miR-3p (brown), and miRNAs not detected in this study (white) in the pre-miRNAs. (B) The miRNAs from 16 pre-miRNAs which show patterns of both miR-5p and miR-3p or either miR-5p and miR-3p present in all ten libraries profiled over pre- and postnatal development stages. Data reflect the most abundant isomiRs scaled based on the total counts of the mappable sequences in each library shown in log_2_-scale. For each miRNA, “New” means miRNA identified in this study; “Diff” means our reported miRNA sequences are different from that of miRBase; “Yes” means our reported miRNA sequences are consistent with those of miRBase. The two miRNAs (miR-1a-3p and miR-122-5p) with the highest expression level (Table S14) are marked by red dots.

A closer look at the mapped known porcine miRNAs from ten libraries in this study indicates that 72 of the 77 known porcine miRNAs and one miRNA* (total 92.3%), were detected (Figure 2A). This high detection rate illustrates that our ten small RNA libraries almost encompass the entire repertoire of previously known miRNAs. Additionally, 36 new miRNA*s have been identified (Table S6). When we looked at the distributions and counts of sequences originating from different arms of the pre-miRNAs, we found that in most cases, counts of sequences were heavily skewed toward the RNA hairpin arm containing the known miRNAs (Figure 2B). In many cases, these low abundance miRNA*s cannot be detected by conventional methods possibly due to their rapid turnover and the spatial, temporal, and physiological regulation of miRNA expression. There is only one known porcine pre-miRNA (mir-140) which codes for two miRNAs. In our experiments, the miR-140-5p (annotated as miR-140) has much lower abundance (in total of 23 counts) than miR-140-3p (annotated as miR-140*, in total of 33 K counts) (Table S6). The miRBase database should be updated to reflect these findings.

The known porcine miRNAs have a broad range of expression levels, varying from millions of sequence counts for the most abundant to single count miRNAs. Twenty-three known porcine miRNAs and two unannotated miRNA*s, corresponding to 22 unique mature miRNA sequences, were detected in all ten libraries (Table S6). Two unique miRNAs, miR-1a-3p and miR-122-5p, known porcine miRNAs, accounted for ∼23.3% and ∼11.2% of the total counts of mappable sequences averaged over all ten libraries, respectively (Figures 1B and 2B (red dots)). miR-1a-3p and miR-122-5p have completely different expression patterns before and after birth, consistent with their well-characterized functions. For miR-1a-3p, a muscle-specific miRNA that promotes myogenesis during embryonic development and muscle cell differentiation [27], [28], the average relative abundance, or counts of the most abundant isomiR normalized against the total counts of the mappable sequences in each library and log_2_-transformed, is −4.51 and −1.12 in pre- and postnatal libraries, respectively. As shown in Table S2, during the four postnatal stages the mixture of samples were abundant with muscle cells: five types of striated muscles and some smooth muscle within the tunica media layer of other tissues. For miR-122-5p, a liver-specific miRNA that has been suggested to play a role in cholesterol, fatty acid and lipid metabolism [29], [30], the average relative abundance is −2.68 and −5.37 in pre- and postnatal libraries, respectively. During the six prenatal stages, the liver makes up a large percentage of whole fetus tissues. Our results correlate well with the fact that in mammals, the rapid morphogenesis of the fetal liver is essential to supporting the developing blood supply [31].

### miRNA's predominant presence as isomiRs

The alignments of the mapped sequ-seqs using known porcine pre-miRNAs as the reference (listed on top of the aligned sequ-seqs) are shown in Table S7. The no-error alignments are listed at the top, followed by one-error aligned sequences. This Table contains 72 queried pre-miRNAs, which were detected in our study and their associated isomiRs for both 5′ and 3′ arms in a total of 6,765 kinds. These statistics for the known porcine miRNAs demonstrated that miRNAs are predominantly present as isomiR families and each isomiR has its own characteristic counts at a given stage of life.

The high definition sequencing used in this study permitted a detailed count of the isomiR sequences which are listed in Tables S7, S9, S11 and S13 by order of abundance (highest to lowest). The kinds of different isomiRs for a given miRNA range from one to 769 (miR-122-5p) and 37 miRNAs exhibit more than one hundred kinds of isomiRs. There are 215 miRNAs (with very low sequence counts, average of 20) that have no isomiRs.

Inspection of Table S7 shows that 40 most abundant isomiR sequences (which takes counts of the ten libraries ≥10) appears not to be the same as that in the miRBase depository. For this comparison, we used enoLOGOS [32] drawings to visualize the sequence heterogeneity patterns as shown in Figure S2. This representation also shows the ratio of counts of the most abundant isomiR and the miRBase entry which ranges from infinitely large (miRBase entry was “not detected”) (such as miR-18-5p, Figure S2A.5) to moderately large (such as let-7i-5p with a ratio of 59,390/352, Figure S2A.1), to very small (such as miR-15b-5p with a ratio of 101/101, Figure S2A.4). For eight of the 40 miRNAs, the miRBase reported sequences were not but the isomiRs were detected.

As shown in Figure S2, the variation patterns of isomiR sequences are quite diverse with the variations primarily concentrated in 3′ positions (32 cases, Figure S2A) as opposed to the 5′ position (four cases, Figure S2B), but variations may also occur at both 5′ and 3′ positions (four cases, Figure S2C). These isomiR sequence variation patterns were also observed in other studies [19], [23], [24]. The presence of isomiRs may have various consequences but the 5′ variations may especially affect the seed sequence which is comprised of the 2^nd^–7^th^ nucleotides of the isomiRs at the 5′ end [5], [33], [34]. The 5′ varied isomiRs might lead to the formation of different miRNA:target mRNA complexes, and thus represent a sort of targeting site which would lead to translational repression.

Closer examination of isomiRs has raised questions concerning miRBase sequences. For example (Figure S2A.1), our sequencing result shows that the most abundant isomiR for let-7i-5p (59,390 counts in all ten libraries) is 22 nt in length. miRBase records ssc-let-7i-5p as a 19 nt sequence which is missing three nucleotides (GUU) at the 3′-end. We found this 19 nt isomiR at 352 counts, which ranked 8^th^ in the isomiR family of let-7i-5p in this study (Table S7). Our result (Figure S3I) is consistent with the finding that this most abundant isomiR (22 nt) is identical to almost all mammalian let-7i-5p in miRBase. Another interesting example relates to miR-103-3p and miR-107-3p (Figure S2A.14 and 16). In miRBase these entries are 23 nt long and have one mismatch at the penultimate nucleotide at the 3′ ends (G-to-C). Our sequencing results reveal the presence of one isomiR (21 nt, deletion of two nt at 3′ ends, 65,719 counts) as the most abundant sequence. Our sequencing experiments did detect these two known porcine miRNAs as the second most abundant miRNAs, but in comparison, the counts are much lower. ssc-miR-103-3p and ssc-miR-107-3p only have 38,201 and 5,437 counts, respectively. Although it is unclear whether the most abundant 21 nt isomiR would function as either or both ssc-miR-103-3p or ssc-miR-107-3p, our results demonstrate the need for documenting isomiRs for the known miRNAs. This documentation is necessary to understand the target-specific regulatory roles of the miRNAome in association with the miRNA variants interaction within the RNA induced silencing complex (RISC).

miRNAs are occasionally observed within the hairpin loop region of pre-miRNA [22], [35], [36]. As part of our study, we searched the sequ-seqs of 1–2 counts and this result is shown in Table S15 with those loop RNAs marked in bold. We found that 27 kinds of sequ-seqs (37 counts) mapped to the hairpin loop regions of 15 pre-miRNAs. Since the lengths of the sequ-seqs matched to the loop regions averaged 19 nt, it is statistically unlikely that these are randomly mapped sequences. Therefore, we observed low, but countable numbers of small RNAs which are located in the loop region. This suggests that the mapped sequ-seqs may be biogenesis intermediate products which are transiently present.

### Mammalian conserved miRNAs in pigs

**Figure 3:**
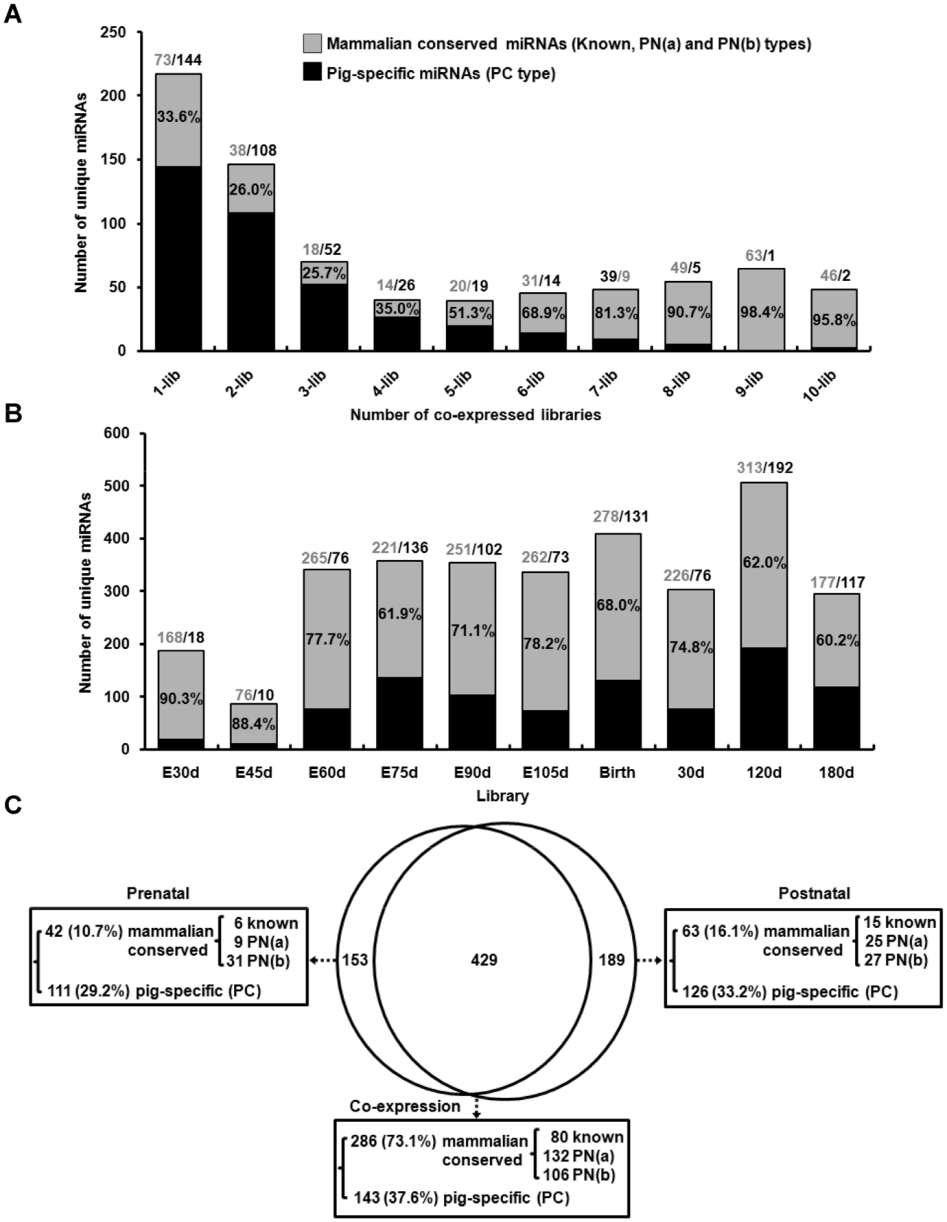
Expressed kinds of 771 unique miRNAs across ten libraries are summarized. (A) Plot of miRNAs detected in one (1-lib), two (2-lib), … 10-libraries (10-lib). The number marked on top of the bar graph shows the miRNAs of two classifications. For example, in 1-lib, 73/144 means that 73 mammalian-conserved miRNAs and 144 pig-specific are present once in the 10 libraries. The bar graphs are depicted in a similar fashion. (B) Plot of miRNAs detected in each sample. The number marked on top of the bar graph shows the miRNAs of two classifications. For example, in E30d, 168/18 means that 168 mammalian-conserved and 18 pig-specific miRNAs were detected in the E30d library. The bar graphs are depicted in a similar fashion. (C) The Venn diagram displays the distribution of 771 miRNAs through the pre- and postnatal stages (note that the classifications of the miRNAs may be overlapping due to assignment of miRNA to more than one kind of categories). Reading from left to right of the Figure shows the distribution of mammalian-conserved miRNAs, to be 42, 286, and 63 sequences (accounting 100%) for prenatal, co-expression, and postnatal stages, respectively; the distribution for pig-specific miRNAs is 111, 143, and 126 sequences (accounting 100%), respectively. The subclasses of the known, PN(a), PN(b) and PC are explained in Table S5.

As shown in Table S14, we classify 493 “PC” (porcine candidate) miRNAs representing 380 unique miRNA sequences derived from genome-mapped sequ-seqs in hairpins and not homologous to any known mammalian miRNAs. These “PC” kinds of miRNAs are likely to be pig-specific. The other 505 miRNAs corresponding to 391 unique miRNA sequences are conserved in mammals. Figures 3 show conserved miRNAs are quite abundant in pig. We examined co-expression of unique miRNAs in ten libraries representing the integrated pre- and postnatal developmental stages. We found that almost all mammalian conserved miRNAs (gray bar-coded in Figure 3A) were ubiquitously expressed in most developmental stages. As shown in Figures 3A, 8- to 10-lib, we can count conserved miRNAs at 49 (out of 54, 90.7%), 63 (out of 64, 98.4%) and 46 (out of 48, 95.8%) unique sequences which were expressed in eight, nine and ten libraries, respectively. Additionally, a larger proportion of the expressed miRNAs (more than 60%) in each library were mammalian conserved miRNAs (gray bar-coded in Figure 3B). Especially during the two early development stages (E30d and E45d), 168 (out of 186, 90.3%) and 76 (out of 86, 88.4%) conserved miRNAs were detected, respectively.

The distributions of sequence counts occurred at 50-fold lower in pig-specific (i.e., PC type) miRNAs (1.5%) than in the known porcine miRNAs and their miRNA*s (75.0%) based on the percent of the counts of mappable sequences (Table S5). It is worth noting that 286 out of 391 (73.1%) conserved unique miRNAs were co-expressed between pre- and postnatal periods (Figure 3C). In contrast, more than half of the 380 non-conserved unique miRNAs (237, 62.4%) appeared to be specific for developmental stages, having expression only in pre- (111, 29.2%) or in postnatal stages (126, 33.2%). These observations in the overall miRNA expression in pig are consistent with reported correlations between conservation of miRNAs and their expression levels [19], [22], [37]. Hypothetically, conserved miRNAs may be responsible for control of the basic cellular and developmental pathways common to most eukaryotes (e.g., cell cycle) whereas the species-specific miRNAs may be involved in regulation of the lineage-specific pathways and functions.

The let-7-family miRNAs, one of the key miRNA regulators in development, are present in abundance across various species including mammals, flies, worms and plants [38]. At present, there are nine members of the let-7-family in mammals: let-7a to -7g, let-7i and let-7j (only identified for dog). We used no-error mapping to differentiate the single base difference in mapping mammalian let-7-family miRNAs, and identified eight let-7-family miRNAs besides let-7j, which were expressed throughout development in all ten libraries (Table S14) and exhibited high levels of sequence conservation in mammals (Figure S3).

### Chromosomal location of pre-miRNAs

**Figure 4:**
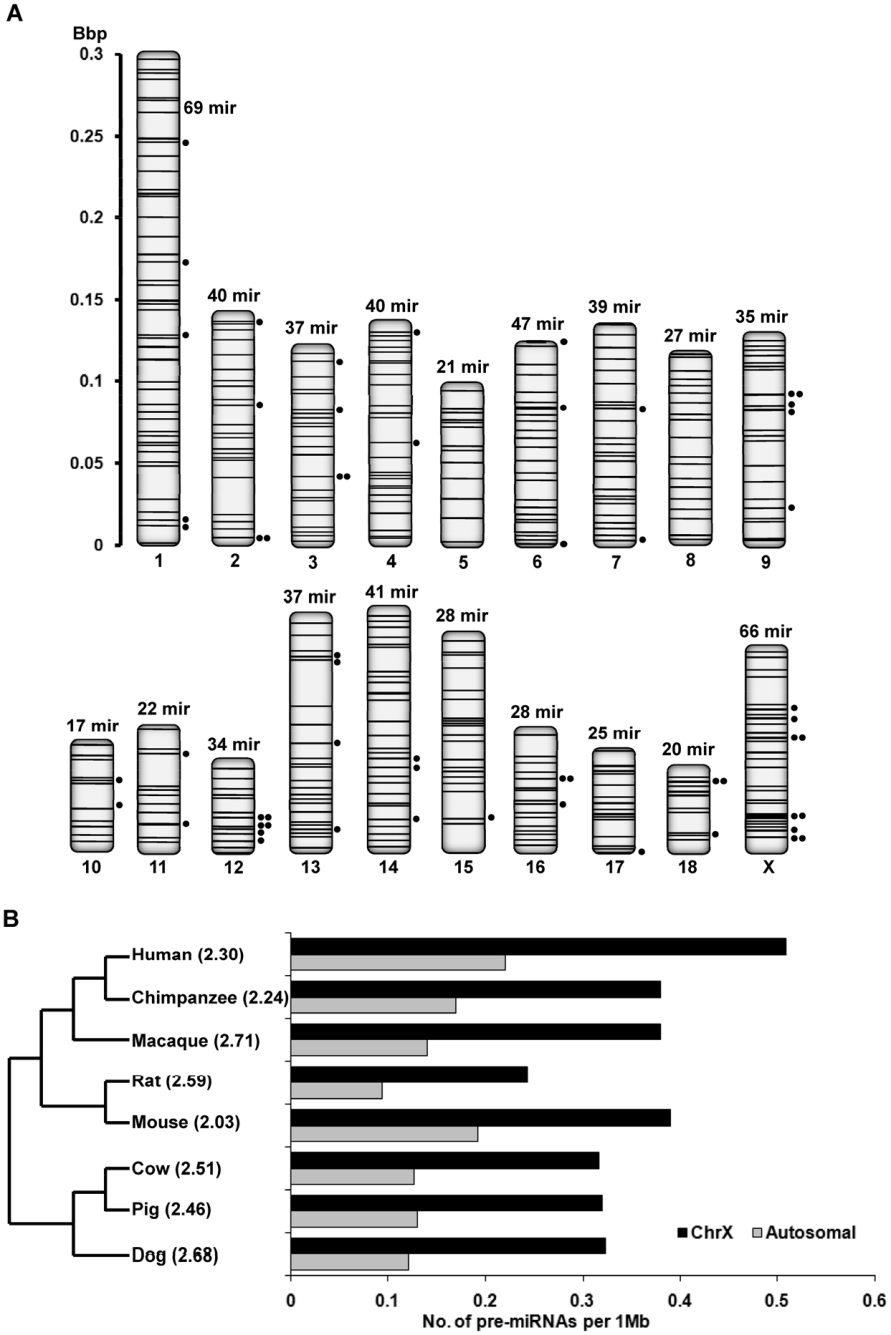
Chromosomal organization of the porcine pre-miRNAs. (A) The chromosomal locations of the porcine pre-miRNAs (mir, black lines) and 59 clusters (under the MID is limited to 50 kb, black dots) are displayed across 19 chromosomes. (B) The comparison of pre-miRNA densities (number of pre-miRNAs per Mb of chromosome) on the X chromosome (dark bar) and autosomes (gray bar) for pig and other seven well-studies mammals is shown pair-wise with the corresponding ratio given in parenthesis. The genome coordinates of pre-miRNAs were derived from miRBase 14.0.

We BLAST searched chromosome positions (Sscrofa9 genome assembly) for all 862 pre-miRNAs as well as five known porcine pre-miRNAs in miRBase that were not detected in our study, and the results are displayed in Figure 4. The search found 623 pre-miRNAs, which accounted for 71.9% of the pre-miRNAs that were compared. We did not observe any pre-miRNA that occurred more than two times in the genome. Fifty pre-miRNAs are located at two genomic loci with 46 of them found on the same chromosome (Table S16). Our analysis found that the genomic density distribution of porcine pre-miRNAs (number of pre-miRNAs per Mb of individual chromosome) is, in general, roughly even (Figure S4G). The shortest chromosome 18 (0.05 Bbp) and the longest chromosome 1 (0.30 Bbp) encode 20 and 69 pre-miRNAs, respectively, corresponding to 0.37 and 0.23 pre-miRNAs for 1 M genome sequences. The X chromosome (medium-length, 0.13 Bbp, ranking 10^th^ in length of the total 19 chromosomes in pig) is an exception in encoding a large number (66 out of 623, 10.6%) of pre-miRNAs, corresponding to 0.52 pre-miRNAs for 1 M genome sequences. The comparison of coefficient of variation (C·V) for the pre-miRNAs densities across all chromosomes for pig and seven well-studied mammals indicated that the distribution of porcine pre-miRNAs may be more dispersive (with the lowest C·V value, 32.5%) than the other seven mammals in miRBase (with an average C·V value, 81.9%) (Figure S4).

Evidence suggesting that miRNAs closely spaced in the genome are co-regulated with host genes has been accumulating [39]. This co-regulation is essential in regulating a complex cell signaling network, and is more efficient than the regulatory pattern mediated by discrete miRNAs [40], [41]. Hence, we grouped pre-miRNAs into clusters (at least two pre-miRNAs in a cluster) if their inter-distance were less than a defined maximum inter-distance (MID) value. Thirty-seven clusters were identified with MID = 3 kb. For MID = 5, 10, or 50 kb, 40, 45, or 59 pre-miRNA clusters were identified (Table S17). In one case, six pre-miRNAs were densely packed within a fragment of ∼800 bps in chromosome 11 (Figure S5).

Reviewing the genomic positions of pre-miRNAs in miRBase 14.0 revealed an average of ∼40.6% (MID  = 3 kb) and ∼51.1% (MID  = 50 kb) of total pre-miRNAs with genome location in human, mouse and rat are likely to cluster. By comparison, the clustering of porcine pre-miRNAs appears less likely as evidenced by the lower percentage of clustering. When MID  = 3 kb, 5 kb, 10 kb or 50 kb, the results are ∼14.8%, ∼15.9%, ∼17.7% or ∼23.9%, respectively when all of the 623 pre-miRNAs were analyzed (Table S17). The four MID groups contain 37, 40, 45 and 59 clusters, respectively, and each group contains 92, 99, 110 and 149 pre-miRNAs. This is due to the incompletely characterized genome in pig. A more detailed analysis of porcine pre-miRNA clusters awaits further development of the pig genome to look for all the hallmarks pre-miRNAs.

### X-linked miRNAs

The XX/XY system is used for sex-determination in the vast majority of mammals. In this study, we note that 66 pre-miRNAs, expressing 71 X-linked miRNAs, out of 623 (10.6%) pre-miRNAs that have genomic coordinates, are located on the X chromosome (Figure 4A). The observed statistics match those reported in the current miRBase for other mammals. For example, based on total known pre-miRNAs those with genome location, 82 (11.4%, human), 64 (11.0%, chimpanzee), 61 (13.5%, macaque), 35 (5.9%, cow), 41 (12.8%, dog), 69 (12.1%, mouse) and 47 (14.8%, rat) are located on the X chromosome. We found a higher density (2.46-fold) of pre-miRNAs on X chromosomes (0.32 pre-miRNAs per Mb of chromosome) than the average of autosomes (0.13 pre-miRNAs per Mb of chromosome) in pig, consistent with that in the other seven mammals (Figure 4B, the phylogenetic tree was modified from Bashir et al. [42]). These X-linked miRNAs were highly expressed in testis [43], [44], and their mRNA targets were exclusively related to the cell cycle process, which is consistent with prolific and continuous cell division during spermatogenesis in the testis [45].

## Discussion

### The high coverage of the sequencing results

We report herein a detailed overview of the experimentally derived pig miRNAome for the first time. Ten small RNA libraries generated a total of 93.6 M sequencing reads, from which 24.0 M counts of mappable sequences (15–26 nt) were derived (Table S5). In all, 88.0% (counts) and 46.4% (kinds) of the mappable sequ-seqs were found to be miRNAs or miRNA candidates (*vide infra*). This high number of redundant sequence counts ensures high quality sequence calls and the coverage of large variations in miRNA expression levels, from a few counts to multiple millions of counts to single digit counts of many miRNAs. This million-fold ratio in measurement greatly expands our view of miRNA expression profile as compared hybridization-based microarray profiling analysis. The process applied layers of filters to remove interfering non-miRNA sequences and provides stringent alignment for matching (zero and one mismatch data is separately aligned thereby allowing independent analysis and data mining of the two groups).

### Pig miRNAome

Our results (Tables S6, S7, S8, S9, S10, S11, S12, S13 and S14) revealed 391 unique porcine miRNAs which are conserved in mammals and 380 unique miRNA candidates likely to be pig-specific. Our data, in combination with miRBase, indicates that the pig miRNAome consists of 1,004 miRNAs originating from 862 pre-miRNAs, of which 777 are sequence-unique miRNAs. In addition, the survey of tissue-specific expression for 30 selected miRNAs raises the possibility that miRNAs identified in our study could be bona fide miRNAs (Figure S6).

The pig miRNAome consists of unevenly distributed counts of sequences in which the top 27 unique miRNAs account for ∼53.5% (by counts) of the mappable sequences versus ∼54.3% determined by the total counts of all 771 unique miRNAs of the mappable sequences in all ten libraries (Table S14). These are considered to be high-count miRNAs whose counts range from 3.1 K (PN(a)95-5p) up to 6.4 M (miR-1a-3p). The high-count miRNAs include the muscle-specific miR-1a-3p, liver-specific miR-122-5p, and the ubiquitous let-7-family miRNAs. Biologically these high-count miRNAs' expression may be more stable, while low-count miRNAs may be prone to perturbation and thus be more responsive to cellular regulatory changes. We note that miRNA distribution would reflect the sample miRNA composition (i.e., the mixture formation from the various tissue samples). The goal of this study was to establish a porcine miRNA atlas for further mapping of tissue- and stage- specific miRNAs. The mapping will reveal the high- and low-count distribution of miRNAs and allow the association of biology with the quantitative presence of specific miRNAs.

The genome scale mapping of miRNAs is a necessary step for revealing the expression, regulation and functions of miRNAs. The nearly completed sequencing of the pig genome allowed us to locate the genomic positions of 623 pre-miRNAs (Figure 4A). These results provide a genomic blueprint for the closely located miRNAs as candidates for co-regulation in their expression and gene targeting.

The miRNAome composition is a dynamic collection of at least hundreds of pre-miRNAs which are processed into mature ∼22 nt RNAs, i.e. miRNAs. Analyzing the sequencing results of the ten libraries that span a pre- and post-natal time period provides information on time-dependent variations of the miRNAome as to (a) sequence lengths, (b) counts and the kinds of miRNAs, and (c) relative expression of conserved versus pig-specific miRNAs. These results have significantly expanded our view on the possible sequences governing the roles of miRNAs.

This work fills a large gap in our knowledge of the pig miRNAome. Only five major publications describe the use of *in silico* predication, conventional or limited deep sequencing methods based on samples of specific categories. In total, these studies reported 298 unique new miRNAs which, thus far, have not been registered in miRBase [46]–[50]. The present study confirms 248 (83.2%) of these sequences (Table S18). The previous studies contributed information concerning the system studied, but left unanswered questions as to miRNAome sequence classification, cataloging, expression profiling, genomic mapping, and temporal miRNAome variation. Our comprehensive set of tissue and time representative samples provides an opportunity to delineate a pig miRNAome atlas.

### miRNA is represented by a family of isomiRs of own abundance

Our deep sequencing survey reveals the details of the pig miRNAome and the isomiRs contained therein. The sequence identity of the isomiR families has broad and significant biological implications. miRNAs have been represented by a single RNA sequence in miRBase, but in reality each miRNA is a family of isomiRs as shown in this and in other studies. Some of these sequences may be generated during sample processing (e.g., sample degradation, RNA breakage, etc.), but there is little doubt that certain isomiRs are signatures of specific biogenesis processes and/or functions, which until now have been largely unidentified. The presence of isomer sequences increases the complexity of the functional roles of these regulatory molecules, but information as provided in this study about precisely the number and identity of the isomiR sequences is requisite for detailed decoding of the miRNAome.

### Concluding remarks

Over last few years, the best studied human and other mammalian miRNAs have revealed rules to discover and define this group of small RNAs of ∼22 nts. Our results show no exceptions for the porcine miRNAs, but this study demonstrates that the definition of miRNAs may require additional parameters. These observations call for more detailed investigations in the pursuit of a thorough understanding of the functional roles of miRNAs. Our studies also reveal unique properties of the pig miRNAome including diverse isomiR sequence patterns and their counts, some of which may be responsible for yet unknown specific functions. Therefore, it is essential to verify whether any of these variants (particularly 5′-end variation) associate within RISC and function as gene silencers. This study provides the most comprehensive list of porcine miRNAs thus far. The availability of such information provides the basis for construction of the genome-scale regulatory networking map of the pig miRNAome. This map has deep and broad implications for protein expression and function in cellular systems, particularly in understanding complex human diseases (e.g., obesity, arthritis and cardiovascular diseases). Equally important, the molecular understanding of pig biology in growth and development will be an important step forward for maximizing the economic benefits in producing high quality pork products for the benefit of human society.

## Materials and Methods

Below are briefly summarized the main features of our methods. Additional details for the process as shown in Figure S1 can be found in Text S1.

### Ethics statement

Rongchang pigs (a white, Chinese indigenous breed) used in this experiment were obtained from the Chongqing Academy of Animal Science, Chongqing, China. Experiments were performed according to the Regulations for the Administration of Affairs Concerning Experimental Animals (Ministry of Science and Technology, China, revised in June 2004) and approved by the Institutional Animal Care and Use Committee in College of Animal Science and Technology, Sichuan Agricultural University, Sichuan, China under permit No. DKY-B20050606. Animals were allowed access to feed and water ad libitum under same normal conditions and were humanely sacrificed as necessary to ameliorate suffering.

### Sample preparation

The ten sample sets of Rongchang pigs used in this experiment are summarized in Table S1. Six sows were artificially inseminated with semen from purebred sires and one sow was humanely sacrificed at each of the six prenatal stages, E30d, E45d, E60d, E75d, E90d and E105d, to obtain three male and three female fetuses. The E30d and E45d fetuses were homogenized after using a PCR-based method for sex determination (Figure S7); other prenatal samples were a mixture of de-boned whole fetus tissues. For four postnatal samples, Birth, 30 d (piglets weaned at 30±1 days), 120 d and 180 d, the mixture samples were derived from 60 tissues (Table S2) with equal amounts of each tissue type. The mixture samples from three individual females and three males were kept separately. The tissue samples were immediately frozen in liquid nitrogen and stored at −80°C.

### Total RNA isolation, small RNA library preparation and sequencing

Total RNA was extracted using *mir*Vana miRNA Isolation Kit (Ambion, Austin, USA) according to the manufacturer's protocol. Subsequently, the sequencing RNA samples were prepared as follows: for each developmental stage, equal quantities (10 µg) of small RNA isolated from six individual pigs were pooled. Approximately 60 µg of small RNA representing each stage of development was used for library preparation and sequencing. Ten libraries were sequenced on the Genome Analyzer GA-I (Illumina, San Diego, USA) following the vendor's recommended protocol for small RNA sequencing.

### Data processing

Sequ-seqs were processed using Illumina's Genome Analyzer Pipeline software and then subjected to a series of data filtration steps, from the statistics of mammalian miRNAs in miRBbase 13.0, to obtain mappable sequences using the ACGT101-miR program.

The merged reference database of pig genome (∼2.26 Bbp) (Sscrofa9, ftp://ftp.sanger.ac.uk/pub/S_scrofa/assemblies/) and non-redundant ESTs (∼0.5 billion nt) (ftp://ftp.ncbi.nih.gov/repository/dbEST) were constructed as an available complete sequence database for pig at present (named as the pig genome and EST) and used for mapping. First, 77 known porcine pre-miRNAs and 3,443 distinct pre-miRNAs from 21 other mammals known from miRBase 13.0 were BLASTed against the mappable sequences in order. Then the mapped pre-miRNAs were further BLASTed against the genome and EST to determined their genomic and EST's locations. The unmapped sequences were BLASTed against the genome and EST, and hairpin RNA structures encompassing sequ-seqs were predicated from the adjacent 60 nt sequences in either direction complying with criteria from the statistics of mammalian pre-miRNAs in miRBbase 13.0 using UNAfold software [51].

### Q-PCR

The expression of 30 selected miRNAs over 45 tissue-specific and two fetal samples were determined by a EvaGreen–based q-PCR approach using a High-Specificity miRNA qRT-PCR Detection Kit (Stratagene, La Jolla, USA) (Table S19).

The data discussed in this publication have been deposited in NCBI's Gene Expression Omnibus and are accessible through GEO Series accession number GSE17885.

## Supporting Information

Figure S1 Analysis workflow and the corresponding supplementary tables and figures.
(2.99 MB TIF)

Figure S2 Sequence logos representing alignments of all isomiRs of the known porcine miRNAs. (A) Thirty-two miRNAs differing in 3′-end alignment. (B) Four miRNAs differing in 5′-end alignment. (C) Four miRNAs differing in both 3′- and 5′-end alignments. In the Figure, the red arrows (for miR-19a-3p, miR-28-5p and miR-301-3p) indicate these sequences are mapped to the genome sequence with one mismatch.
(8.42 MB TIF)

Figure S3 Sequence alignments of the sequenced let-7-family miRNAs and the homologous mammalian let-7 family miRNAs. (A) The alignment of the eight let-7-family miRNAs identified in our study. “Total count” is the sum for the most abundant isomiR in all ten libraries. These sequences share identical seed sequence (2nd to 7th from the 5′ end, 5′-GAGGTA-3′). The alignments of each sequenced let-7 miRNAs with the corresponding mammalian let-7-family of miRNAs: (B) PN(a)1-1-5P (as same sequence as PN(a)1-2-5p) aligned to homologous let-7a; (C) PN(a)2-5p aligned to homologous let-7b; (D) ssc-let-7c-5p (known porcine miRNA) aligned to the corresponding miRBase ssc-let-7c; (E) PN(a)3-5p aligned to homologous let-7d; (F) PN(a)4-5p aligned to homologous let-7e; (G) ssc-let-7f-5p (known porcine miRNA) aligned to the corresponding miRBase ssc-let-7f; (H) PN(a)6-5p aligned to homologous let-7g; (I) ssc-let-7i-5p (known porcine miRNA) aligned to the corresponding miRBase ssc-let-7i. bta: Bos taurus, cfa: Canis familiaris, eca: Equus caballus,hsa: Homo sapiens, mdo: Monodelphis domestica, mml: Macaca mulatta, mmu: Mus musculus, oan: Ornithorhynchus anatinus, ptr: Pan troglodytes, rno: Rattus norvegicus, ssc: Sus scrofa.
(7.38 MB TIF)

Figure S4 Densities of pre-miRNAs on chromosomses for pig and other seven well-studies mammals. Densities were calculated by dividing the number of pre-miRNAs on the individual by the length of nucleotides on the corresponding chromosome (shown in left brackets), which shown as number of pre-miRNAs per megabase of DNA. The densities across all chromsomes are also shown as Mean ± SD, the coefficient of variation (C•V%) are also given at the upper right corner of each plot. The genome coordinates of pre-miRNAs on chromosomes were from miRBase 14.0. The plots are (A) human (Homo sapiens, GRCh37), (B) chimpanzee (Pan troglodytes, CHIMP2.1), (C) macaque (Macaque mulatta, MMUL1.0), (D) rat (Rattus norvegicus, RGSC 3.4), (E) mouse (Mus musculus, NCBIM37), (F) cow (Bos Taurus, BTAU4.0), (G) pig (Sus scrofa, Sscrofa9) and (H) dog (Canis familiaris, CanFam 2.0).
(9.35 MB TIF)

Figure S5 Pre-miRNA hairpin cluster structure containing six porcine pre-miRNAs. The gene cluster is located on the sense strand of chromosome 11 and the coordinate information is: mir-17: 36,340,189–36,340,268; mir-18: 60,972,593–60,972,684; mir-19a: 60,972,741–60,972,822; mir-20: 60,972,911–60,972,981; PN(a)46-1: 60,973,037–60,973,123; PN(a)119-1 : 60,973,159–60,973,236.
(2.80 MB TIF)

Figure S6 Q-PCR analysis of expression of 30 selected unique miRNAs (24 are previously unannotated miRNAs) across 47 samples. Data shown are log2-transformed of the relative expression amount. Six pairs of two miRNAs originating from a same pre-miRNA are denoted by a bracket, the corresponding counts of most abundant isomiR in all ten sequencing librariies are also listed at bottom.
(7.31 MB TIF)

Figure S7 Sex determination of porcine fetuses at day 30 and 45 using SRYB/STS-Bol PCR duplex PCR system. Females in lanes 1 to 6 and 8, males in lanes 7, 9 and 10. Lane M, DNA size marker. Upper band (163 bp) corresponds to the SRYB PCR product and is present only in males. Lower band (326 bp) corresponds to the positive control STS-Bol PCR product and is present in all pigs. The band sizes (bottom to top) of DL2000 DNA marker (TaKaRa) are 2,000, 1,000, 750, 500, 250 and 100 bp.
(2.98 MB TIF)

Table S1 Sample sets information in this study.
(0.02 MB XLS)

Table S2 Catalog of 60 types tissues mixed for four postnatal sample sets (Birth, 30 d, 120 d and 180 d).
(0.03 MB XLS)

Table S3 Counts and kinds of low (counts <3) vs. high-copy (counts ≥3) sequenced sequences (sequ-seqs).
(0.02 MB XLS)

Table S4 Distribution of counts and kinds of sequ-seqs originating from known RNA classes in different libraries.
(0.03 MB XLS)

Table S5 Statistics based on the counts and kinds of the mappable sequences.
(0.03 MB XLS)

Table S6 Profile of the known porcine miRNAs (miRBase 14.0).
(0.09 MB XLS)

Table S7 The alignments of isomiRs for known porcine pre-miRNAs (miRBase 14.0).
(1.95 MB XLS)

Table S8 Profile of novle miRNAs originating from pre-miRNAs (PN(a) type) that can be mapped to pig genome or EST.
(0.17 MB XLS)

Table S9 The alignments of isomiRs for pre-miRNAs (PN(a) type) that can be mapped to pig genome or EST.
(2.38 MB XLS)

Table S10 Profile of novle miRNAs originating from pre-miRNAs (PN(b) type) that can not be mapped to pig genome or EST.
(0.16 MB XLS)

Table S11 The alignments of of isomiRs for pre-miRNAs (PN(b) type) that can not be mapped to pig genome or EST.
(0.90 MB XLS)

Table S12 Profile of candidate miRNAs originating from predicted RNA hairpins (PC type pre-miRNAs).
(0.32 MB XLS)

Table S13 The alignments of isomiRs for predicted RNA hairpins (PC type pre-miRNAs).
(1.01 MB XLS)

Table S14 Porcine unique miRNAs.
(0.35 MB XLS)

Table S15 Atypical pattern of miRNA processing.
(0.52 MB XLS)

Table S16 Porcine pre-miRNAs with two genome locations.
(0.03 MB XLS)

Table S17 Genome location clusters of porcine pre-miRNAs.
(0.06 MB XLS)

Table S18 Comparison of new identified miRNA between other reports and our discovery.
(0.08 MB XLS)

Table S19 Primer sequences of the q-PCR experiments.
(0.02 MB XLS)

Text S1 Supplementary methods. Includes methods and supporting results not discussed directly in the text.
(0.10 MB DOC)
