# Supplementary figures and images for: MicroRNAome of Porcine Pre- and Postnatal Development
Source: PLoS One. 2010 Jul 12;5(7):e11541. doi: 10.1371/journal.pone.0011541 (PMC2902522; doi:10.1371/journal.pone.0011541)

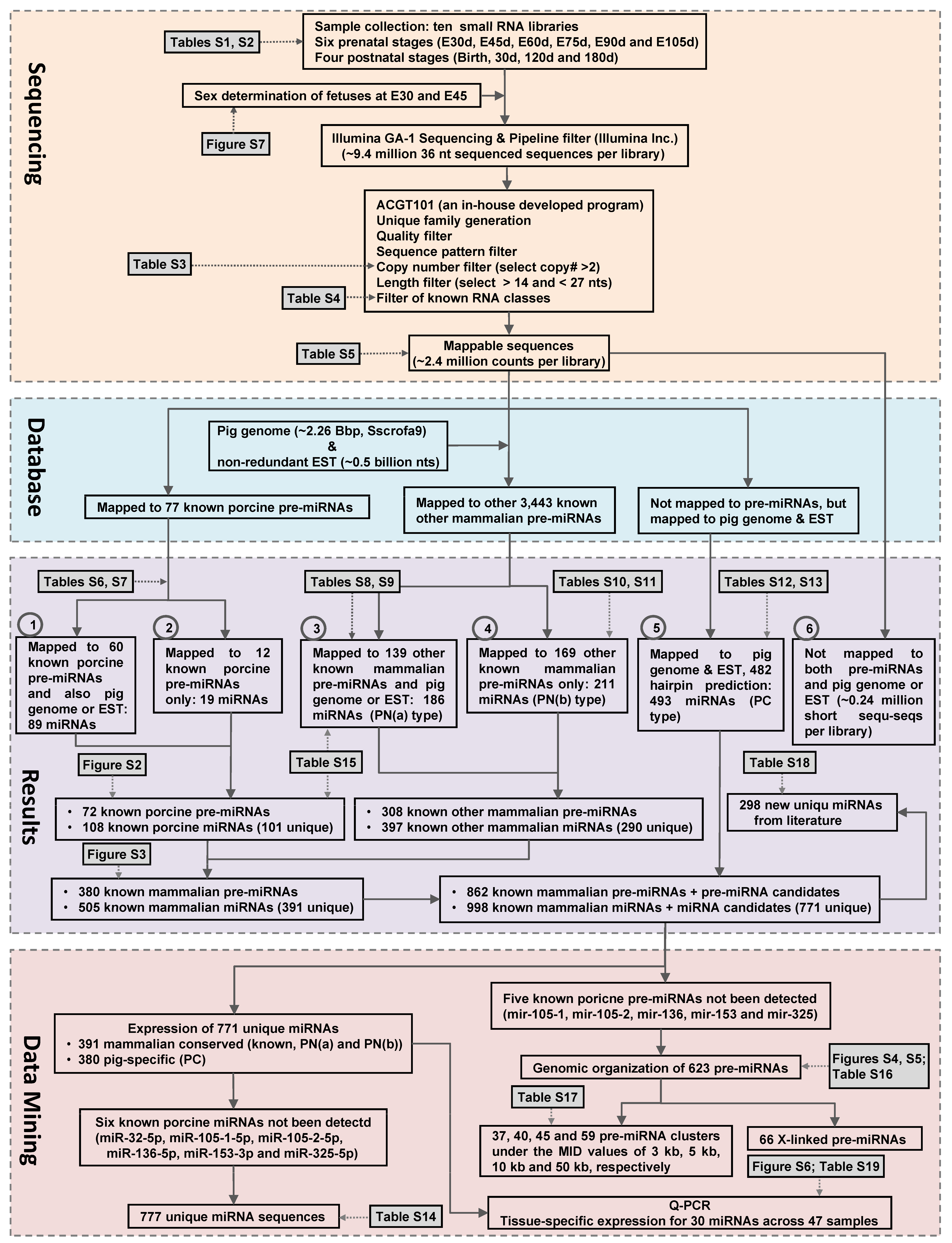

Supplement: Figure S1 — Analysis workflow and the corresponding supplementary tables and figures. (2.99 MB TIF) [file pone.0011541.s001.tif]

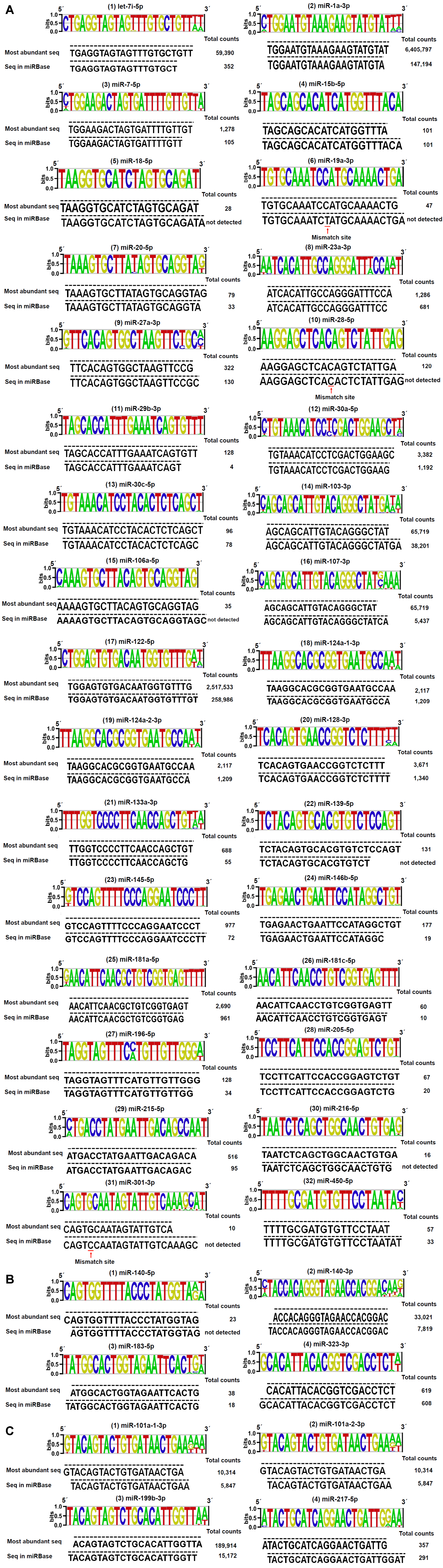

Supplement: Figure S2 — Sequence logos representing alignments of all isomiRs of the known porcine miRNAs. (A) Thirty-two miRNAs differing in 3′-end alignment. (B) Four miRNAs differing in 5′-end alignment. (C) Four miRNAs differing in both 3′- and 5′-end alignments. In the Figure, the red arrows (for miR-19a-3p, miR-28-5p and miR-301-3p) indicate these sequences are mapped to the genome sequence with one mismatch. (8.42 MB TIF) [file pone.0011541.s002.tif]

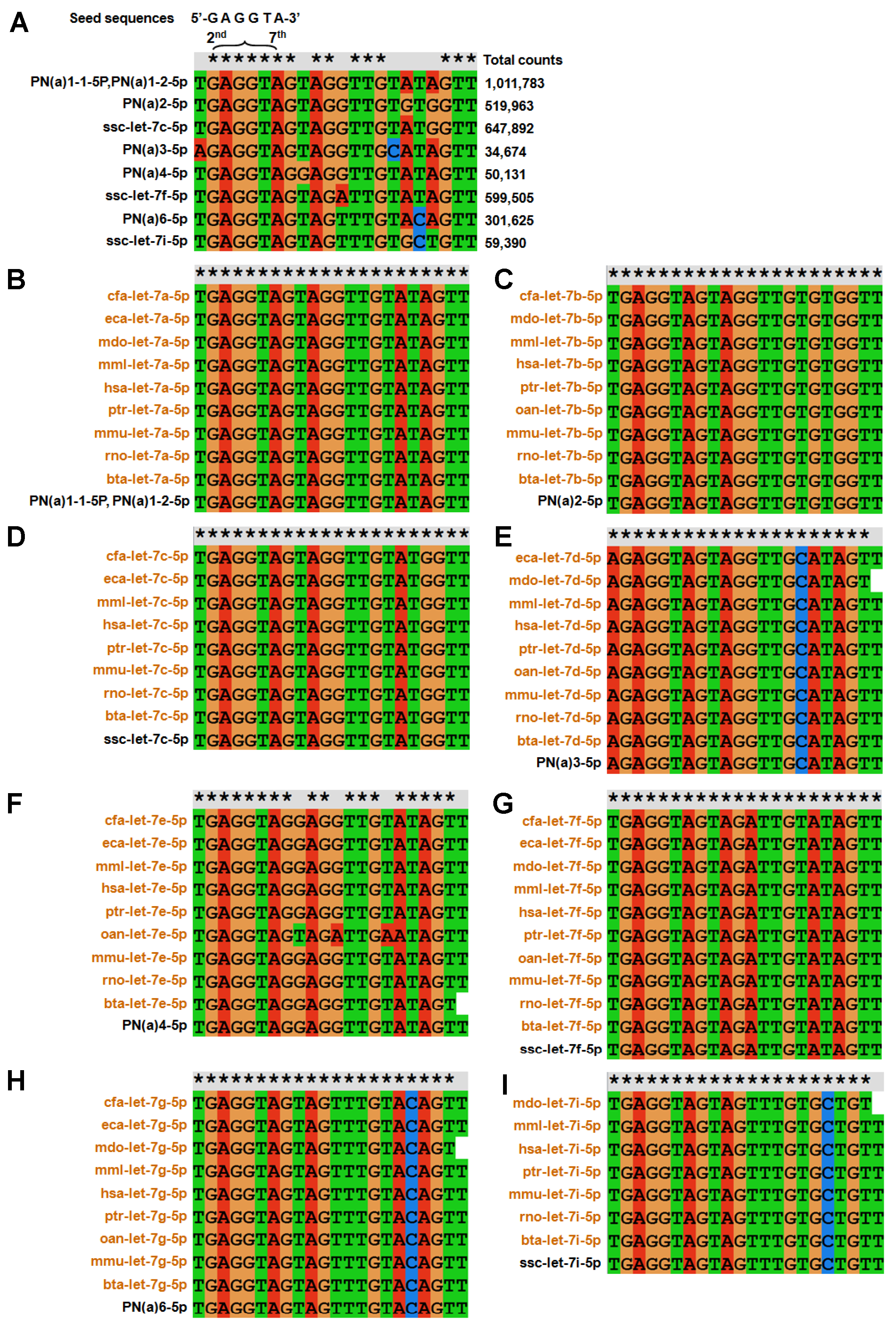

Supplement: Figure S3 — Sequence alignments of the sequenced let-7-family miRNAs and the homologous mammalian let-7 family miRNAs. (A) The alignment of the eight let-7-family miRNAs identified in our study. “Total count” is the sum for the most abundant isomiR in all ten libraries. These sequences share identical seed sequence (2nd to 7th from the 5′ end, 5′-GAGGTA-3′). The alignments of each sequenced let-7 miRNAs with the corresponding mammalian let-7-family of miRNAs: (B) PN(a)1-1-5P (as same sequence as PN(a)1-2-5p) aligned to homologous let-7a; (C) PN(a)2-5p aligned to homologous let-7b; (D) ssc-let-7c-5p (known porcine miRNA) aligned to the corresponding miRBase ssc-let-7c; (E) PN(a)3-5p aligned to homologous let-7d; (F) PN(a)4-5p aligned to homologous let-7e; (G) ssc-let-7f-5p (known porcine miRNA) aligned to the corresponding miRBase ssc-let-7f; (H) PN(a)6-5p aligned to homologous let-7g; (I) ssc-let-7i-5p (known porcine miRNA) aligned to the corresponding miRBase ssc-let-7i. bta: Bos taurus, cfa: Canis familiaris, eca: Equus caballus,hsa: Homo sapiens, mdo: Monodelphis domestica, mml: Macaca mulatta, mmu: Mus musculus, oan: Ornithorhynchus anatinus, ptr: Pan troglodytes, rno: Rattus norvegicus, ssc: Sus scrofa. (7.38 MB TIF) [file pone.0011541.s003.tif]

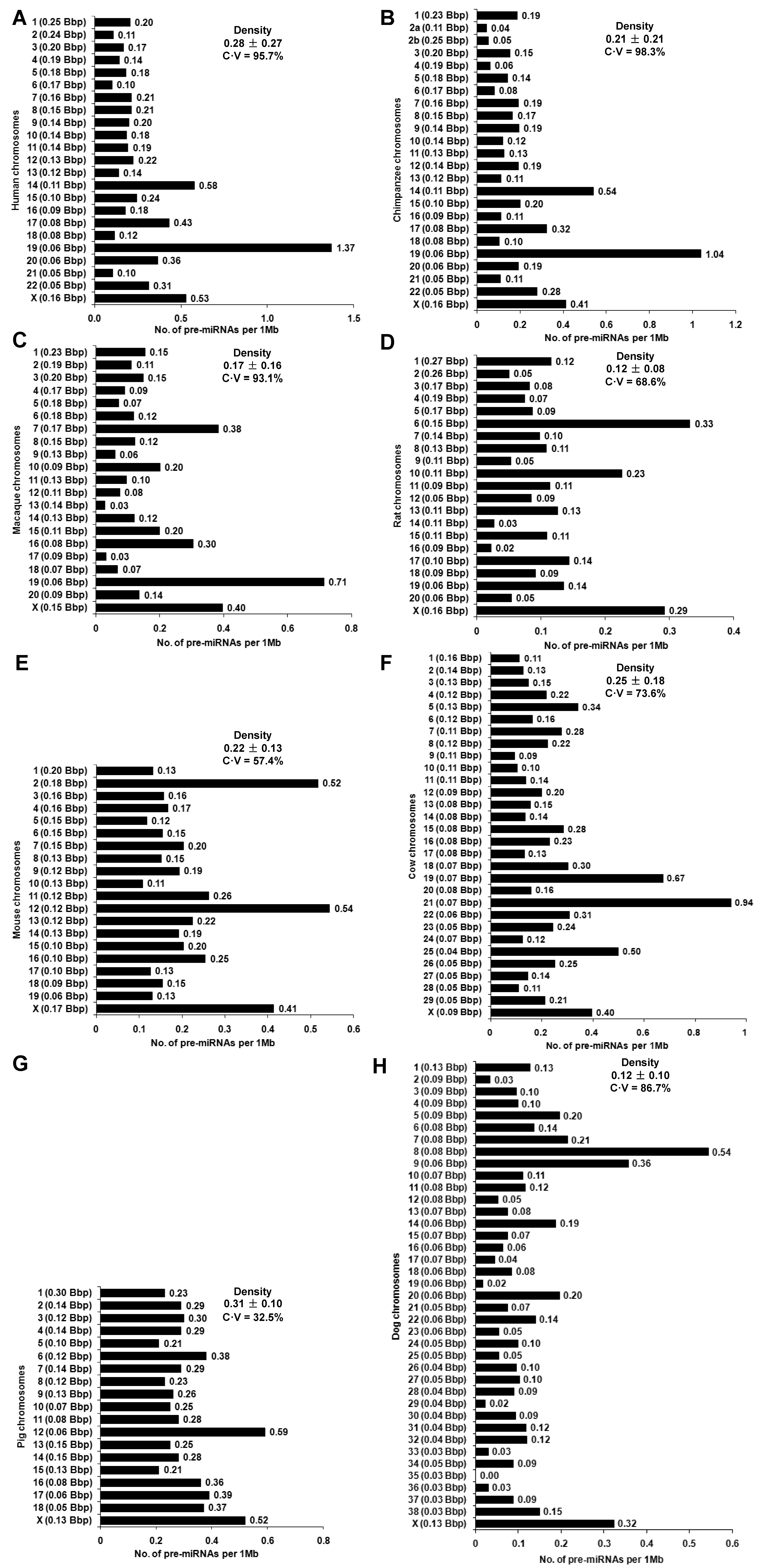

Supplement: Figure S4 — Densities of pre-miRNAs on chromosomses for pig and other seven well-studies mammals. Densities were calculated by dividing the number of pre-miRNAs on the individual by the length of nucleotides on the corresponding chromosome (shown in left brackets), which shown as number of pre-miRNAs per megabase of DNA. The densities across all chromsomes are also shown as Mean ± SD, the coefficient of variation (C•V%) are also given at the upper right corner of each plot. The genome coordinates of pre-miRNAs on chromosomes were from miRBase 14.0. The plots are (A) human (Homo sapiens, GRCh37), (B) chimpanzee (Pan troglodytes, CHIMP2.1), (C) macaque (Macaque mulatta, MMUL1.0), (D) rat (Rattus norvegicus, RGSC 3.4), (E) mouse (Mus musculus, NCBIM37), (F) cow (Bos Taurus, BTAU4.0), (G) pig (Sus scrofa, Sscrofa9) and (H) dog (Canis familiaris, CanFam 2.0). (9.35 MB TIF) [file pone.0011541.s004.tif]

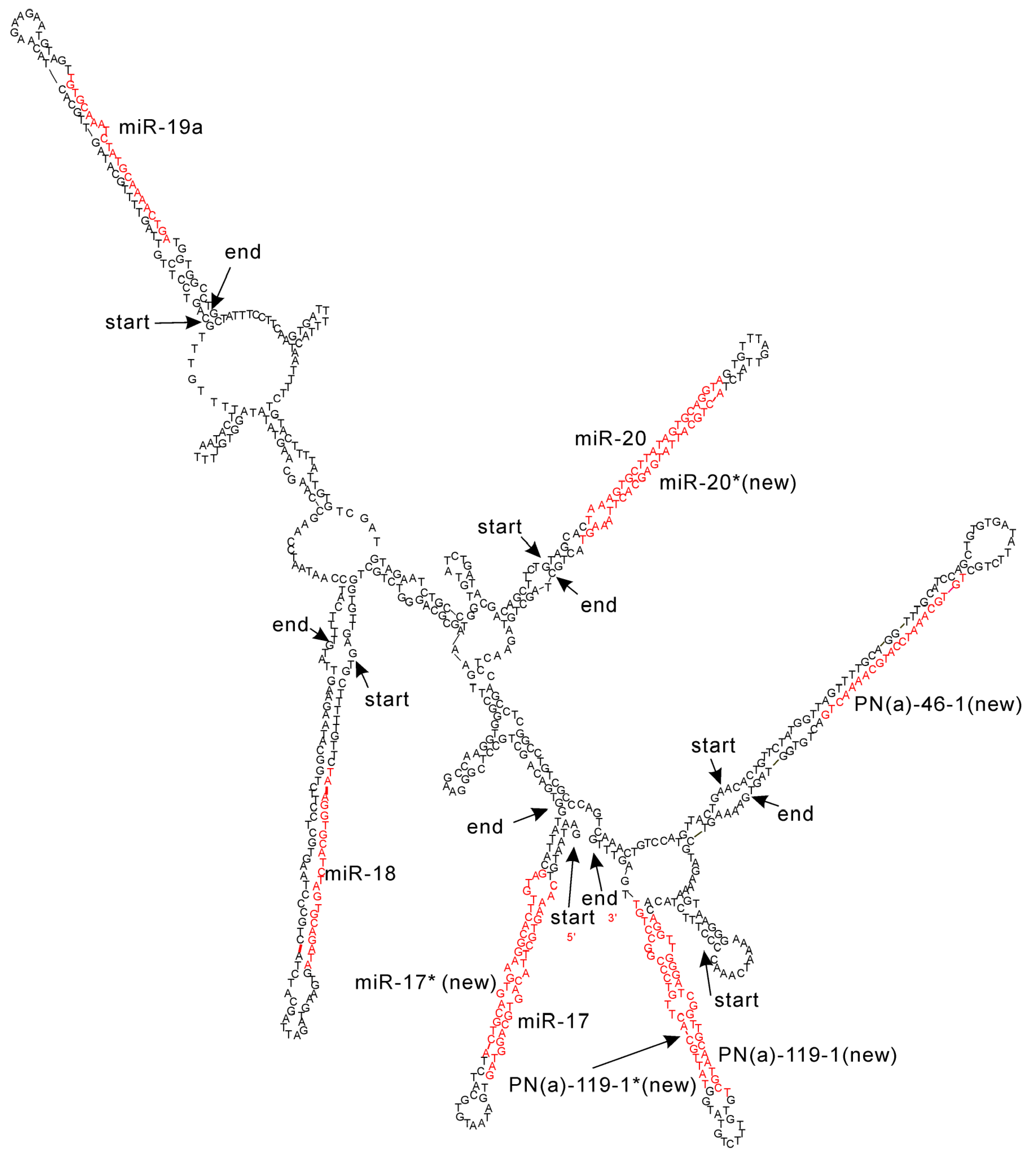

Supplement: Figure S5 — Pre-miRNA hairpin cluster structure containing six porcine pre-miRNAs. The gene cluster is located on the sense strand of chromosome 11 and the coordinate information is: mir-17: 36,340,189–36,340,268; mir-18: 60,972,593–60,972,684; mir-19a: 60,972,741–60,972,822; mir-20: 60,972,911–60,972,981; PN(a)46-1: 60,973,037–60,973,123; PN(a)119-1 : 60,973,159–60,973,236. (2.80 MB TIF) [file pone.0011541.s005.tif]

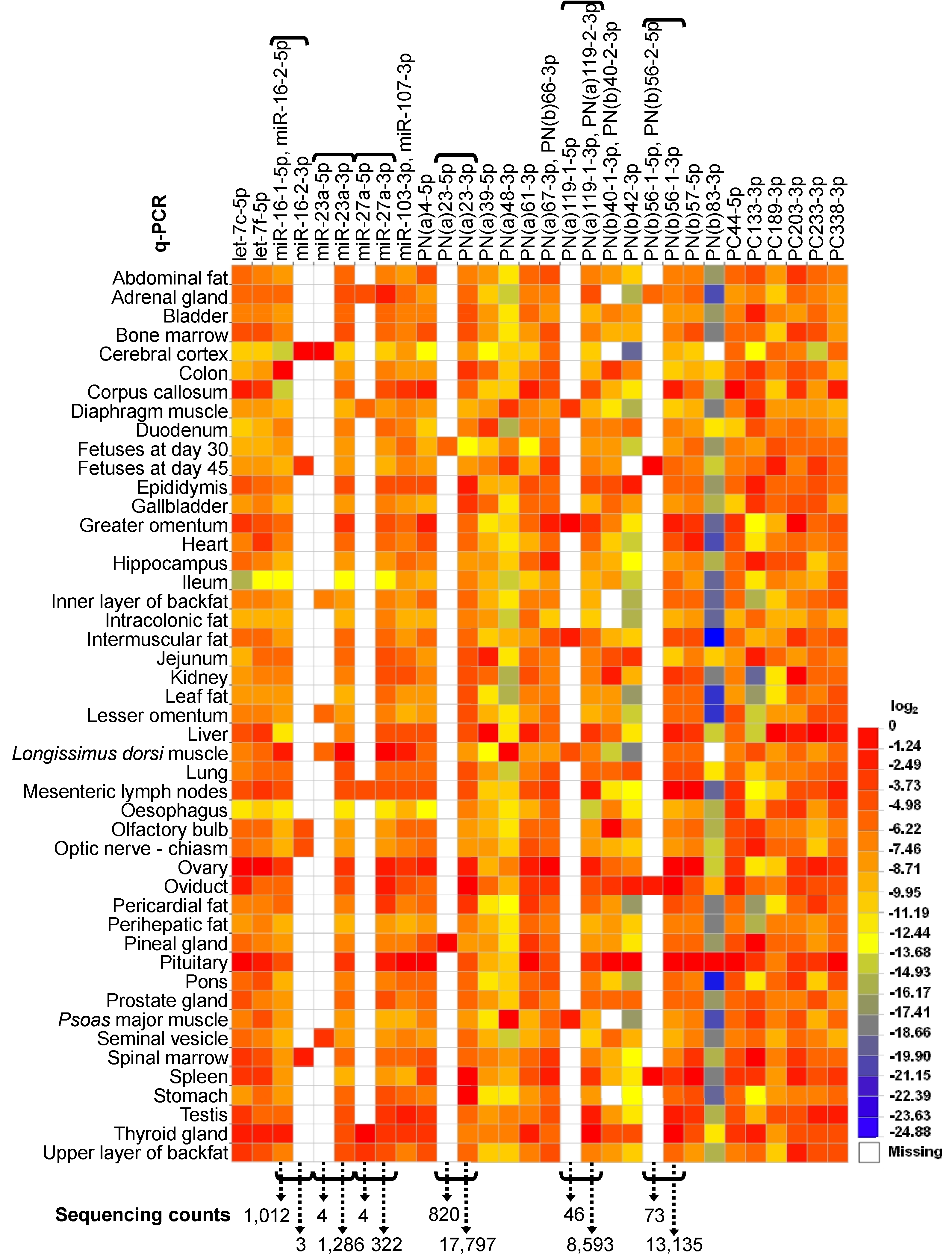

Supplement: Figure S6 — Q-PCR analysis of expression of 30 selected unique miRNAs (24 are previously unannotated miRNAs) across 47 samples. Data shown are log2-transformed of the relative expression amount. Six pairs of two miRNAs originating from a same pre-miRNA are denoted by a bracket, the corresponding counts of most abundant isomiR in all ten sequencing librariies are also listed at bottom. (7.31 MB TIF) [file pone.0011541.s006.tif]

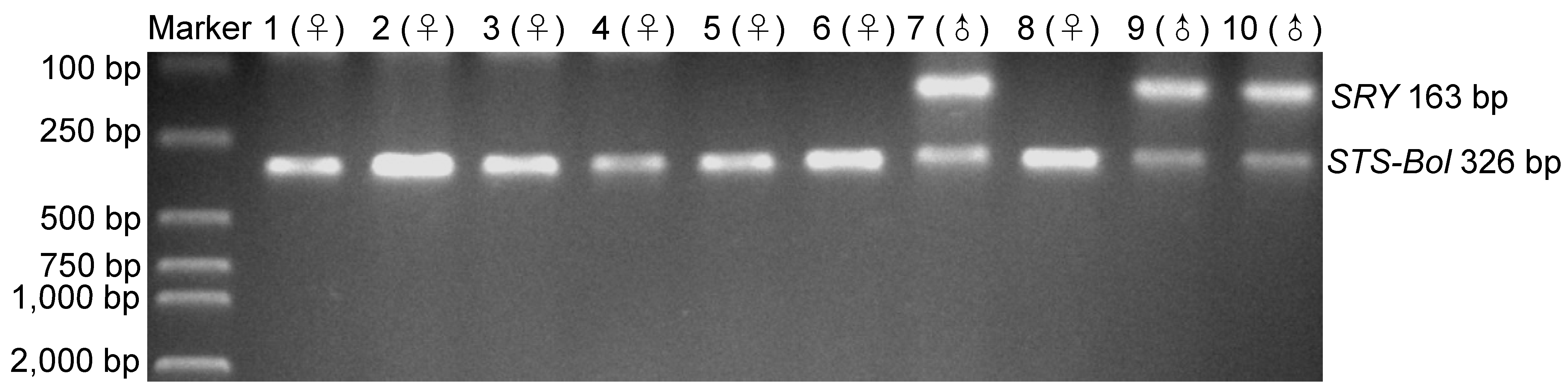

Supplement: Figure S7 — Sex determination of porcine fetuses at day 30 and 45 using SRYB/STS-Bol PCR duplex PCR system. Females in lanes 1 to 6 and 8, males in lanes 7, 9 and 10. Lane M, DNA size marker. Upper band (163 bp) corresponds to the SRYB PCR product and is present only in males. Lower band (326 bp) corresponds to the positive control STS-Bol PCR product and is present in all pigs. The band sizes (bottom to top) of DL2000 DNA marker (TaKaRa) are 2,000, 1,000, 750, 500, 250 and 100 bp. (2.98 MB TIF) [file pone.0011541.s007.tif]
