# Supplementary material for: MicroRNAome of Porcine Pre- and Postnatal Development
Source: PLoS One. 2010 Jul 12;5(7):e11541. doi: 10.1371/journal.pone.0011541 (PMC2902522; doi:10.1371/journal.pone.0011541)
Supplement: Text S1 — Supplementary methods. Includes methods and supporting results not discussed directly in the text. (0.10 MB DOC) [file pone.0011541.s027.doc]

**Supplementary Methods:**

Figure S1 illustrates the overall workflow of our study, from sample collection to final report of the pig microRNAome.

## Sample preparation

Rongchang pigs with ad libitum access to feed under the same normal conditions were used in this study. The animals were reared in compliance with national regulations for the humane care and use of animals in research. Total RNA samples were collected from six prenatal stages (30, 45, 60, 75, 90 and 105 days after insemination) and four postnatal stages (birth, 30, 120 and 180 days). Samples were collected from entire fetuses or mixtures of overwhelming majority tissues. The detailed information is provided in Tables S1 and S2.

***Sample sets of six prenatal stages*:** Six pregnant sows were used for fetal samples. Immediately after exsanguination of a sow at each time point, the uteri were recovered from which ~9-12 fetuses (theoretically, half male and half female) were quickly removed and processed as described below. To obtain the overwhelming majority of tissues/cell types that represent the whole pig body, we individually homogenated each entire fetus at day 30 and 45 after insemination, and mixed all the abdominal organs, reproductive organs, brain, eyes, tongue, et al., and the commensurable muscle, fat and skin, only discard the bones for day 60, 75, 90 and 105 after insemination. Equal quantities (10 µg) of small RNA isolated from three male and three female fetuses or pigs were pooled to make up each sample. Approximately 60 µg of small RNA representing each developmental stage was used for library preparation for sequencing.

***Sex determination of fetuses at day 30 and 45*:** The sexual characteristics of pig fetuses were not visible before day 49 of the post-conception period. Therefore, the gender of fetuses at day 30 and 45 was determined using a PCR-based method as previously described [1] with minor modifications [2]. Only after sex determination was a sequencing sample set of three male and three female fetuses constructed.

Briefly, a fragment of the sex-determining region Y (*Sry*) located on the Y chromosome was amplified using the primers SRYB-3 and SRYB-5 along with the autosomal locus STS-Bo1 (sequence tagged site-Bo1) [3] serving as an internal control (Figure S7). The 5’ upstream primer (SRYB-5) was 5’-TGAACGCTTTCATTGTGTGGTC-3’ and the 3’ downstream primer (SRYB-3) was 5’-GCCAGTAGTCTCTGTGCCTCCT-3’ to amplify a 163 bp region of *Sry* (GenBank accession: U49860). The 5’ upstream primer (STS-Bo1-5) was 5’-gagcagactccaactactctcactccac-3’ and the 3’ downstream primer (STS-Bo1-3) was 5’-tcagaaggatgatttagagtgtctgttcag-3’ to amplify a 326 bp fragment as a simple codominant marker STS-Bo1 (GenBank accession: AF450118). The genomic DNA was isolated from fetal tissues using the QIAamp DNA Micro Kit (Qiagen). The DNA was amplified using the Taq PCR Master Mix Kit (Qiagen) in 10 µM containing 100 ng of genomic DNA, 12.5 µl Taq PCR Master Mix，0.4 pmol·µl^-1^ forward and reverse primer each, add RNase-free H_2_O to total volume 25 µl. The cycling parameters used were as follows: 95 °C for 4 min; 35 cycles of 95 °C for 15 s, 60 °C for 45 s, 72 °C for 30 s; and 72 °C for 5 min.

***Sample sets of four postnatal stages*:** Twenty-four pigs were used for mixed tissues samples. The piglets were weaned simultaneously at day 30 ± 1. We mixed 60 normal tissues from three males and three females at each time point in equal amounts for each type of tissue (~0.05 g in birth, ~0.1 g in day 30, and ~0.2 g in day 120 and 180) (Table S2).

## Total RNA Isolation, Small RNA Library Preparation and Sequencing

The low molecular weight RNA was extracted using *mir*Vana miRNA Isolation Kit (Ambion) according to the manufacturer’s protocol. The total RNA quantity and purity passed the analysis of NanoDrop ND-1000 spectrophotometer (Nano Drop) at 260/280 nm (ratio > 2.0). The integrity of total RNA also passed the analysis of Agilent Bioanalyzer 2100 and RNA 6000 Nano LabChip Kit (Agilent) with RIN number > 6.0.

In brief, the sequencing was performed as follows: the small RNA fraction between 10-40 nt was isolated by polyacrylamide gel electrophoresis (PAGE) and ligated with proprietary adaptors (Illumina). The short RNAs were then converted to cDNA by RT-PCR and the cDNA was sequenced on the Genome Analyzer GA-I (Illumina) following the vendor’s recommended protocol for small RNA sequencing.

**Data processing**

The analysis workflow for sequencing data is outlined in Figure S1.

***Sequencing reads from Pipeline software*:** First, the raw sequenced sequences (sequ-seqs) were subjected to the Illumina Pipeline filter provided by the vendor (Solexa 0.3). The quality of a specific base call was determined using either the chastity or the purity of the base. Chastity is the ratio of the brightest intensity over the sum of the brightest and the second brightest intensities. Purity is the ratio of the brightest intensity over the sum of all four (A, C, G, or T) intensities at each sequencing cycle for the first 25 cycles. The pipeline filtered the sequ-seqs according to the relation "failed-chastity ≤ 1", using a chastity threshold of 0.6, where failed-chastity is the cycle with lower than threshold chastity. This removed all sequ-seqs with chastity less than 0.6 for two or more cycles among the first 25 cycles. This indicates that there may be one cycle with chastity less than 0.6 among the first 25 cycles. This cycle may have an ambiguous base call, since the brightest and the second brightest intensities are very close to each other. This also means that allowing one mismatch is reasonable when mapping sequences to pre-miRNA sequences or the pig genome because one base call may have uncertainty.

***Obtaining “mappable sequences”* :** Each of the pipeline filter treated data sets was further processed with an in-house program called ACGT101-miR (to be published separately). The sequ-seqs that passed through a series of additional filters are called “mappable sequences”, which are sequences satisfy the following criteria from the statistics of mammalian miRNAs in miRBbase 13.0: (1) not sequencing adapters; (2) containing no more than 80% A, C, G, or T; (3) containing no more than two N (undermined bases); (4) containing no stretches of A_7_, C_8_, G_6_, or T_7_; (5) been observed more than two times; (6) being longer than 14 nt and shorter than 27 nt; and (7) not originating from known classes of RNAs (i.e., mRNA, rRNA, tRNA, snRNA, snoRNA and repetitive sequence elements).

The “known classes of RNAs” filter is used to remove sequences which could not be miRNA candidates. These are (a) mRNAs (from NCBI by searching the nucleotide database for '*Sus Scrofa* mRNA' on March 18, 2009; the sequence files with file names starting with “NM” and “XM” were used), (b) rRNA and other kinds of RNAs of known cellular functions (from Rfam at ftp://ftp.sanger.ac.uk/pub/databases/Rfam/9.1 on March 18, 2009, including rRNA, tRNA, snRNA and snoRNA sequences; but excluding the sequences whose sequence ID contains the words 'MIR', 'mir', 'let' or ‘lin’) [4], and (c) repetitive sequences (from Repbase sequences downloaded from http://www.girinst.org/server/archive/ on March 18, 2009) [5].

***Reference databases used for mapping sequ-seqs*:** The mappable sequences or transcripts were mapped with several reference databases as shown in Figure S1 and as discussed below.

(a) miRBase pig (ssc) and other mammalian miRNAs and pre-miRNAs

The mammalian mature miRNA sequences (miRs) and pre-miRNA hairpin gene sequences (mirs) are recorded in miRBase 13.0, and are conserved across species in sequence. This information was used as a reference of known miRNAs and pre-miRNAs for mapping the sequ-seqs. The database thus far contains 4,753 mature miRNAs (2,266 are unique) that correspond to 4,327 pre-miRNA hairpin genes (3,520 are unique) in a total of 22 mammals. Among them, there are 78 known porcine miRNAs (73 are unique) (ssc-miRs) which correspond to 77 known porcine pre-miRNA hairpin genes (ssc-mirs). miRNAs and pre-miRNAs are conserved in other species.

(b) Pig genome and ESTs

While the pig genome size is estimated to be about 2.7 billion base pairs (Bbp), the latest draft genome assembly of the pig (Sscrofa9, May 2009) produced by the Wellcome Trust Sanger Institute was only 2.26 Bbp long. We also constructed an EST database containing more than one million “non-redundant EST” sequences with regard to the pig genome (over 0.51 billion nt) as shown in the Table below. The pig genome and EST sequences have a total size of ~2.7 Bbp plus 0.51 billion nt.

Pig ESTs were selected from an EST depository (ftp://ftp.ncbi.nih.gov/repository/dbEST, October 30, 2008) if the identity of an EST contained “ssc” and the length of the EST sequence was greater than or equal 50. After removing duplicates, the unique EST sequences were BLASTed against the pig genome (Sscrofa9, ftp://ftp.sanger.ac.uk/pub/S_scrofa/assemblies/). If the sequence similarity (based on full length of EST sequence) between the EST sequences and genome was equal to or less than 80%, the EST sequence was considered a non-redundant EST (See Table below). We updated the sequence information by incorporating the non-redundant EST and pig genome sequence (Sscrofa9) as described. The resulting reference database (referred as genome and EST) was used to map sequ-seqs (i.e., mappable sequences).

**Table. Summary of pig** EST data

| **Type** | **Number of EST** | **Number of nucleotides** |
| --- | --- | --- |
| Total EST | 1,475,183 | 668,253,678 |
| Unique EST | 1,395,507 | 659,302,114 |
| Non-redundant EST | 1,044,849 | 514,304,284 |

***Mappable sequences mapped to miRBase miRNAs and to pig genome and EST*:** As shown in Figure S1, the 3,520 (77 in pig and 3,433 in other mammals) unique pre-miRNAs in miRBase 13.0 were BLASTed against mappable sequences of less than 27 nt. Pre-miRNAs were considered as “mapped” if the number of errors (including deletion, insertion and mismatch) was equal to or less than one. The “mapped pre-miRNAs” were subsequently BLASTed against the genome and EST. The “mapped pre-miRNAs” were considered as “mapped to the pig genome and EST” if the sequence similarity (based on full length of pre-miRNAs sequence) of the mapped pre-miRNAs and the genome and EST was equal to or greater than 90%.

The first group contained 72 out of 77 (~94%) known porcine pre-miRNAs, of which 60 could be mapped to both mappable sequences and the genome and EST. The remaining 12 pre-miRNAs could only be mapped to mappable sequences but not to the genome and EST (Tables S6 and S7).

The mapped pre-miRNAs in other mammals (excluding pig) were further classified into two categories: (1) pre-miRNAs that were mapped to mappable sequences and the genome and EST, and temporarily named PN(a) (porcine novel, “a” type), and (2) pre-miRNAs that were mapped to mappable sequences but not to the genome and EST, temporarily named PN(b) (porcine novel, “b” type). Within the PN(a) category, pre-miRNAs were clustered according to their genomic locations. The pre-miRNAs that are located within a distance of 50 nt are considered to be within the same cluster, i.e., position-based cluster. Within the PN(b) category, pre-miRNAs were clustered according to their sequences since these pre-miRNAs do not map to the genome and cannot be allocated to a genomic position. This is based on the BLAST computation. If the sequ-seq similarities among the miRNAs (not the pre-miRNAs) are equal to or greater than 90%, or 0.9 length coverage for program NCBI-BLASTClust, the corresponding pre-miRNA sequences are grouped into one sequence cluster, i.e., sequence-based cluster. These results are shown in Tables S8 and S9 for the PN(a) category and Tables S10 and S11 for the PN(b) category.

***RNA hairpin structure prediction*:** The mappable sequences which were not mapped to known mammalian pre-miRNAs were subjected to mapping to genome and EST sequences. The mapping allowed one error anywhere or two errors if the additional error was located at the 3’-terminal (the 30^th^ base and beyond) since the Illumina sequencer has higher base calling error rates in this region. For RNA hairpin structure prediction, the genomic coordinates from above miRNA mapping were extended by 60 nt towards both their 5’ and 3’ terminals. The corresponding genomic sequences (extended genomic sequences) were retrieved and used as an input for the UNAfold software. The maximum folding energy (ΔG_max_ = -15 kCal·mol^-1^) used for the presence of a hairpin structure was obtained from the folding energy calculated for mammalian pre-miRNAs in miRBase 13.0. For each retrieved genomic sequence, the hairpin folding was evaluated by sequence analysis to identify the presence of a stem with equal to or more than 16 base pairs and the folding energy was calculated using UNAfold software. The final set of hairpin sequences reported in Tables S12 and S13 are those having folding energy equal to or less than ΔG_max_.

More specifically, the criteria used include, (1) number of base pairs in a stem ≥ 16 bp; (2) number of allowed errors in one bulge ≤ 18; (3) free energy (ΔG) ≤ -15 kCal·mol^-1^; (4) percentage of miRNA appearing in the stem ≥ 80%; (5) length of hairpin (up and down stems plus hairpin loop) ≥ 53 nt; and (6) length of hairpin loop ≤ 22 nt. If a sequence satisfies these strict criteria, this sequence is considered as a predicted pre-miRNA candidate. These criteria were applied to known mammalian pre-miRNA sequences, and overall 93.4% of 4,327 pre-miRNA sequences qualify for these criteria (See Table below).

**Table. Application of** the six criteria to known mammalian pre-miRNAs sequences

| **ID** | **Species** | **# of qualified pre-miRNAs** | **# of known pre-miRNAs** | **%** |
| --- | --- | --- | --- | --- |
| 1 | age (*Ateles geoffroyi*) | 58 | 60 | 96.7 |
| 2 | bta (*Bos taurus*) | 335 | 356 | 94.1 |
| 3 | cfa (*Canis familiaris*) | 294 | 321 | 91.6 |
| 4 | cgr (*Cricetulus griseus*) | 1 | 1 | 100.0 |
| 5 | ggo (*Gorilla gorilla*) | 83 | 86 | 96.5 |
| 6 | hsa (*Homo sapiens*) | 657 | 706 | 93.1 |
| 7 | lca (*Lemur catta*) | 14 | 16 | 87.5 |
| 8 | lla (*Lagothrix lagotricha*) | 46 | 48 | 95.8 |
| 9 | mdo (*Monodelphis domestica*) | 115 | 119 | 96.6 |
| 10 | mml (*Macaca mulatta*) | 431 | 463 | 93.1 |
| 11 | mmu (*Mus musculus*) | 512 | 547 | 93.6 |
| 12 | mne (*Macaca nemestrina*) | 72 | 75 | 96.0 |
| 13 | oan (*Ornithorhynchus anatinus*) | 292 | 331 | 88.2 |
| 14 | oar (*Ovis aries*) | 3 | 4 | 75.0 |
| 15 | pbi (*Pygathrix bieti*) | 11 | 11 | 100.0 |
| 16 | ppa (*Pan paniscus*) | 88 | 89 | 98.9 |
| 17 | ppy (*Pongo pygmaeus*) | 82 | 84 | 97.6 |
| 18 | ptr (*Pan troglodytes*) | 541 | 594 | 91.1 |
| 19 | rno (*Rattus norvegicus*) | 280 | 286 | 97.9 |
| 20 | sla (*Saguinus labiatus*) | 39 | 42 | 92.9 |
| 21 | ssc (*Sus scrofa*) | 76 | 77 | 98.7 |
| 22 | ssy (*Symphalangus syndactylus*) | 11 | 11 | 100.0 |
| Sum | | 4,041 | 4,327 | 93.4 |

The predicted pre-miRNAs, temporarily identified as “PC” for porcine candidate, were clustered according to their genomic locations. Pre-miRNAs in the same cluster are those located within 50 nt. The predicted pre-miRNAs were derived from the extended genomic sequences (60 nt from sequ-seqs). From the statistics of 706 known human hairpins, on average, the 5’ and 3’ terminals of hairpins away from the stem region contain three nucleotides. Therefore, we allowed three nucleotides to overhang the terminals from the stem regions of predicted hairpins. But if the sequences exceeded the stem plus the overhang of three nucleotides, the overhang was extended to cover the sequences. The outside parts from the overhangs on both sides were cut away.

## Q-PCR

Total RNA was extracted using the *mir*Vana Kit (Ambion) from 47 diverse samples, including (1) entire fetuses at day 30 and 45 collected for the sequencing analysis and (2) 41 normal tissues from three 120 day old female Rongchang pigs, and four additional male tissues were reproductive tissues (epididymis, testis, prostate and seminal vesicle) from three 120 day old male Rongchang pigs. The forward primers of 30 selected unique miRNAs were identical in sequence and length to the miRNA itself (i.e., the most abundant isomiR) based on our sequencing results, which are available in Table S19. The EvaGreen–based q-PCR [6] was performed with the High-Speciﬁcity miRNA qRT-PCR Detection Kit (Stratagene) on the iQ5 Real-Time PCR Detection System (Bio-Rad). The ΔΔCt method was used to determine the expression level differences between surveyed samples. All measurements contained a negative control and a no-*E. coli* poly A polymerase (PAP) control, and each sample of each individual was analyzed in triplicate. Normalized factors (NF) of three internal control genes (U6 snRNA, 18S rRNA and Met-tRNA) and relative quantities of objective miRNAs were analyzed using qBase software [7].

## Tissue-specific expression of selected miRNAs

The Figure S6 is a q-PCR profiling map based on the experiments carried out as validation for selected miRNAs identified in the Illumina sequencing. This set of experiments analyzed 30 selected unique miRNAs in 45 specific tissues and two time points (E30d and E45d). Most of them were ubiquitously expressed in almost all tissues analyzed, but six unique miRNAs, miR-16-2-3p, miR-23a-5p, miR-27a-5p, PN(a)23-5p, PN(a)119-1-5p and PN(b)56-1-5p (as same sequence as PN(b)56-2-5p), which exhibited the low expression, belong to six pairs of two mature miRNAs originating from one pre-miRNA. These q-PCR results are consistent with the statistics of the counts of the most abundant isomiR in sequencing approach. The relative abundances clearly indicate these six miRNAs are secondarily expressed miRNAs to their respective predominant one (from the opposite arm of the pre-miRNA), and should be designated as miRNA*s.

**Supplementary References:**

1. Pomp D, Good BA, Geisert RD, Corbin CJ, Conley AJ (1995) Sex identification in mammals with polymerase chain reaction and its use to examine sex effects on diameter of day-10 or -11 pig embryos. J Anim Sci 73: 1408-1415.

2. Murani E, Muraniova M, Ponsuksili S, Schellander K, Wimmers K (2007) Identification of genes differentially expressed during prenatal development of skeletal muscle in two pig breeds differing in muscularity. BMC Dev Biol 7: 109. doi: 10.1186/1471-213X-7-109.

3. Wimmers K, Murani E, Ponsuksili S, Yerle M, Schellander K (2002) Detection of quantitative trait loci for carcass traits in the pig by using AFLP. Mamm Genome 13: 206-210.

4. Gardner PP, Daub J, Tate JG, Nawrocki EP, Kolbe DL, et al. (2009) Rfam: updates to the RNA families database. Nucleic Acids Res 37: D136-140.

5. Jurka J, Kapitonov VV, Pavlicek A, Klonowski P, Kohany O, et al. (2005) Repbase Update, a database of eukaryotic repetitive elements. Cytogenet Genome Res 110: 462-467.

6. Mao F, Leung WY, Xin X (2007) Characterization of EvaGreen and the implication of its physicochemical properties for qPCR applications. BMC Biotechnol 7: 76. doi: 10.1186/1472-6750-7-76.

7. Hellemans J, Mortier G, De Paepe A, Speleman F, Vandesompele J (2007) qBase relative quantification framework and software for management and automated analysis of real-time quantitative PCR data. Genome Biol 8: R19. doi: 10.1186/gb-2007-8-2-r19.
